# Correlation between changes in functional connectivity in the dorsal attention network and the after-effects induced by prism adaptation in healthy humans: A dataset of resting-state fMRI and pointing after prism adaptation

**DOI:** 10.1016/j.dib.2018.12.053

**Published:** 2018-12-18

**Authors:** Kengo Tsujimoto, Katsuhiro Mizuno, Daisuke Nishida, Masatoshi Tahara, Emi Yamada, Shiori Shindo, Yuuki Watanabe, Shoko Kasuga, Meigen Liu

**Affiliations:** a Department of Rehabilitation Medicine, Keio University School of Medicine, 35 Shinanomachi, Shinjuku-ku, Tokyo 160-8582, Japan; b Saiseikai Kanagawaken Hospital, 6-6 Tomiyacho, Kanagawa, Yokohama, Kanagawa Prefecture 221-8601, Japan; c Center for Neuroscience Studies, Queen׳s University, Botterell Hall, 18 Stuart Street, Kingston, Ontario K7L 3N6, Canada; d Japan Society for Promotion of Science, Kojimachi Business Center Building, 5-3-1 Kojimachi, Chiyoda-ku, Tokyo 102-0083, Japan

**Keywords:** Prism adaptation, Resting-state functional connectivity, Attention network, Unilateral spatial neglect, Sensorimotor learning

## Abstract

It has been reported that it is possible to observe transient changes in resting-state functional connectivity (FC) in the attention networks of healthy adults during treatment with prism adaptation. by using functional magnetic resonance imaging (fMRI) (see “Prism adaptation changes resting-state functional connectivity in the dorsal stream of visual attention networks in healthy adults: A fMRI study” (Tsujimoto et al., 2018) [1].

Recent neuroimaging and neurophysiological studies support the idea that prism adaptation (PA) affects the visual attention and sensorimotor networks, which include the parietal cortex and cerebellum.

These data demonstrate the effect of PA on resting-state functional connectivity between the primary motor cortex and cerebellum. Additionally, it evaluates changes of resting-state FC before and after PA in healthy individuals using fMRI. Analyses focus on FC between the primary motor cortex and cerebellum, and the correlation between changes in FC and its after-effects following a single PA session. Here, we show data that demonstrate the change in resting-state FC between the primary motor cortex and cerebellum, as well as a correlation between the change ratio of FC and the amplitude of the after-effect.

**Specifications table**

**Table:** 

Subject area	Brain imaging
More specific subject area	Functional magnetic resonance imaging, Prism adaptation
Type of data	Figures
How data were acquired	1.5-T MR scanner (Signa Excite, Optima MR450w 1.5T, GE Healthcare, United Kingdom)
Data format	Analyzed data
Experimental factors	These data consisted of healthy adults
Experimental features	Resting-state: participants kept their eyes open and fixated on a black cross. Each resting-state scan lasted for 10 min.
Data source location	Shinjuku-ku, Tokyo, Japan
Data accessibility	Data are within this article
Related research article	Tsujimoto K, Mizuno K, Nishida D, Tahara M, Yamada E, Shindo S et al. Prism adaptation changes resting-state functional connectivity in the dorsal stream of visual attention networks in healthy adults: A fMRI study. Cortex, 18, pp. 30351–30354, 2018.

**Value of the data**

• This dataset demonstrates rapid changes in resting-state functional connectivity between the primary motor cortex and the cerebellum as a result of prism adaptation in healthy adults observed using fMRI.
• This dataset demonstrates a correlation between the change ratio of FC and the amplitude of the after-effect.
• This study will contribute to the understanding of the underlying mechanism of prism adaptation and will improve the treatment of unilateral spatial neglect (USN) by PA therapy.

## 1 Data

**Fig. 1:**
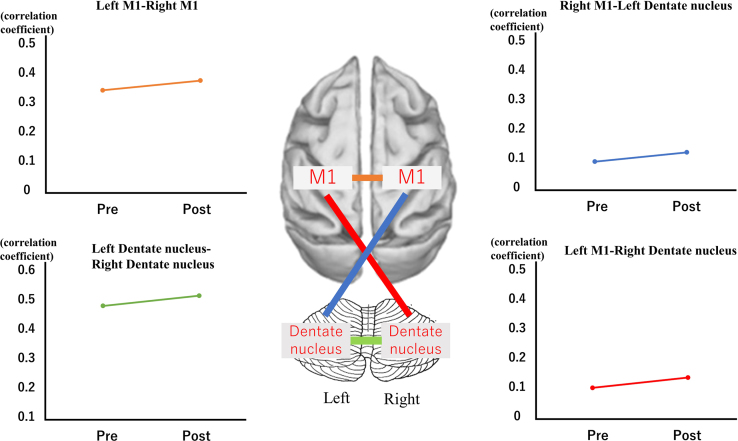
Functional connectivity can be observed between the right primary motor cortex and the left primary motor cortex, and between the right dentate nucleus and the left dentate nucleus.

The data represent a detailed characterization of the relationship between the primary motor cortex and the cerebellum by prism adaptation in healthy adults using fMRI (Fig. 1).

**Fig. 2:**
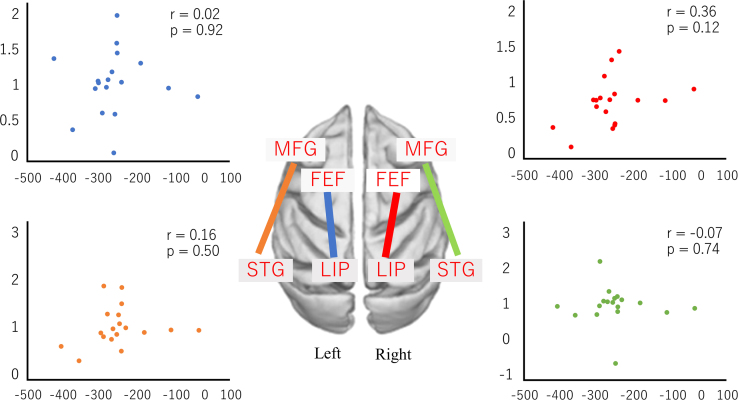
Correlation between the change ratio of FC and the amplitude of after-effect in dorsal and ventral attention networks.

**Fig. 3:**
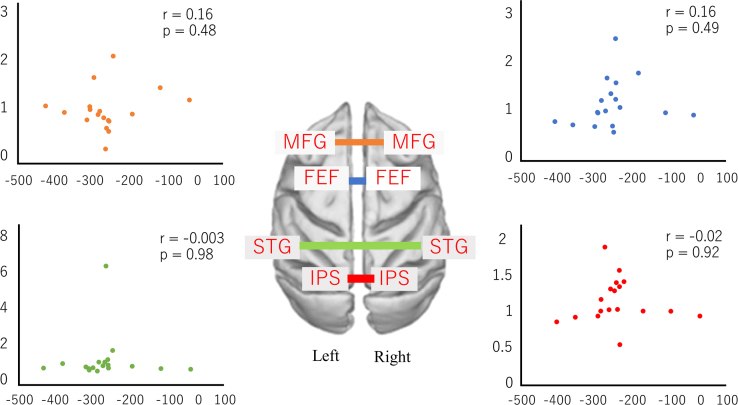
The relationship between the attention network areas of the right and left sides of the brain.

**Fig. 4:**
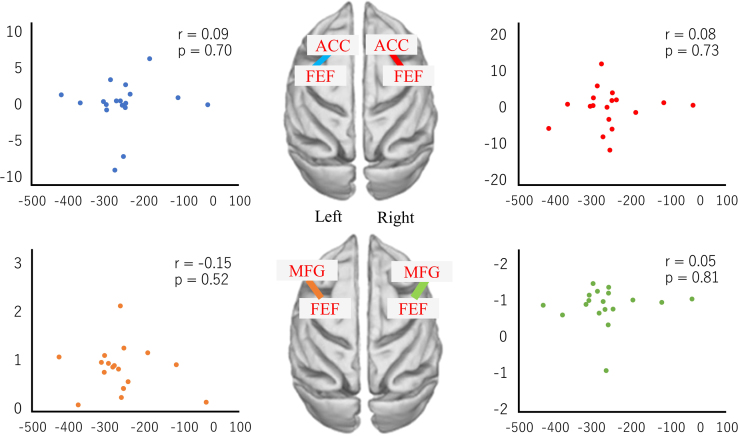
The correlation between change ratio of FC and the amplitude of after-effect in the dorsal attention network.

**Fig. 5:**
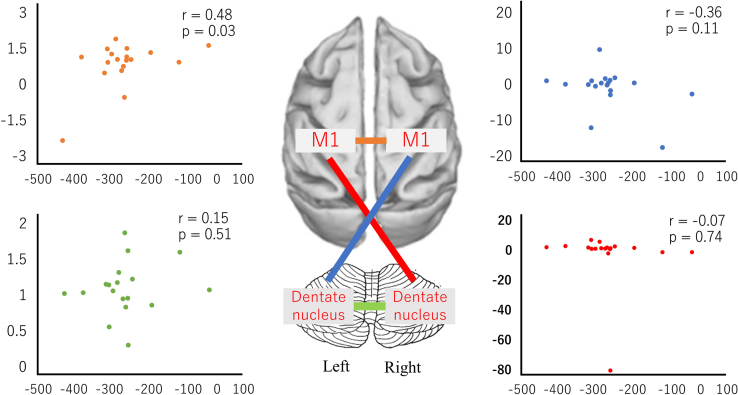
Correlation between the change ratio of FC and the amplitude of after-effect among bilateral primary motor cortex and dentate nucleus.

The data represent correlation between the change ratio of FC and the amplitude of after-effect (Fig. 2, Fig. 3, Fig. 4, Fig. 5).

## 2 Experimental design, materials, and methods

The group statistical analysis included a total of 19 healthy participants (average age 28.5 ± 5.5; 8 males and 11 females).

A bar-shape target with a length of 15 cm and a width of 3 mm was displayed on a 25 in. touch panel display (PL2452MT, Mouse_Computer, Co., Ltd, Nagoya, Japan). The target was randomly presented at 3 locations (body midline; 3.75 cm right and left of the midline). Participants were wearing prism glasses that shift visual field to the right by 20 diopters. At the baseline, participants performed six pointing movements without a prism. In the prism adaptation (PA) phase, 90 pointing movements were performed wearing prism glasses. At the post PA phase, six pointing movements were performed without prism glasses. At the baseline and in the PA phase, the distance between the touch panel display and the desk was 5 cm. Participants were able to see their finger at the end of movements. In the post PA phase, the distance between the desk and display was 0 cm. Therefore, the participant could not see their finger at the end of movements in this phase.

The fMRI data described here were recorded with a 1.5-T MR scanner (Signa Excite, Optima MR450w 1.5T, GE Healthcare, United Kingdom), during resting state (10 min.) and before and after the PA session. Regions that were activated during the PA session in previous studies were selected as regions of interest (ROIs) [2], [3], [4], [5]. ROIs included the primary motor cortex (M1) (±39, 18, 49) and Cerebellum (Dentate Nuclei) (±14, 54, −23). Preprocessing of the fMRI data was performed using Analysis of Functional NeuroImages (AFNI) ver.16.0.11 [6] (https://afni.nimh.nih.gov/afni/). The first five volumes of each fMRI scan were discarded due to unstable magnetization. Thereafter, 8800 functional volumes corrected for the slice timing differences in each volume, head movement, and the image drift between sessions. Spatial smoothing with a 3 mm Gaussian kernel and bandpass time filtering between 0.01 and 0.1 Hz were applied.

## 3 FC between M1 and cerebellum

We tested FC between the M1 and cerebellum (dentate nuclei). The two correlation coefficients were converted to a normally distributed z-score by Fisher׳s transformation Thereafter, they were averaged and used for ANOVA with Bonferroni correction for multiple comparison (Fig. 1).

## 4 Correlation between the change ratio of FC and the amplitude of after-effect

To evaluate the after-effect of the participants in the pointing tasks, pointing errors were recorded immediately after the PA session. Lateral displacement of the movement endpoints relative to the respective target was measured in cm for each pointing movement. The after**-**effect was defined as the average of the pointing errors in the six trials after the PA session.

We calculated the ratio relating FC before the PA session to the FC after the PA session and evaluated the correlation between the change ratio of FC and amplitude of after**-**effect. Pearson correlation coefficients were analyzed using MATLAB (R 2015b). *P* < 0.05 was considered statistically significant (Fig. 2, Fig. 3, Fig. 4, Fig. 5).

It can also be observed between the right primary motor cortex and the left dentate nucleus, and between the left primary motor cortex and the right dentate nucleus. The red line indicates the functional connectivity between the left M1 and the right dentate nucleus. The blue line indicates the functional connectivity between the right M1 and the left dentate nucleus. The green line indicates the functional connectivity between the right dentate nucleus and the left dentate nucleus. The orange line indicates the functional connectivity between the right M1 and the left M1. Vertical axes indicate the correlation coefficient values. Horizontal axes indicate the experimental phases. (See Fig. 1.)

The blue dots indicate the change ratio of FC between left frontal eye field (FEF) and left IPS. The red dots indicate the change ratio of FC between right FEF and left IPS. The orange dots indicate the change ratio of FC between the left MFG and left STG. Finally, the green dots indicate the change ratio of FC between the right MFG and right STG. Vertical axes indicate the change ratio of FC. Horizontal axes indicate the amplitude of after**-**effect. (See Fig. 2.)

The blue dots indicate the change ratio of FC between the left FEF and the right FEF. The red dots indicate the change ratio of FC between the right IPS and the left IPS. The orange dots indicate the change ratio of FC between the left MFG and the left MFG. Finally, the green dots indicate the change ratio of FC between the right STG and the left STG. Vertical axes indicate the change ratio of FC. Horizontal axes indicate the amplitude of after**-**effect. (See Fig. 3.)

Fig. 4 shows the correlation between change ratio of FC and the amplitude of after**-**effect in the following pairs: the right frontal eye field and right anterior cingulate cortex; the left eye frontal field and the left anterior cingulate cortex; the right middle frontal gyrus and the right frontal eye field; and the left middle frontal gyrus and left frontal eye field. The blue dots indicate the change ratio of FC between the left ACC and the right FEF. The red dots indicate the change ratio of FC between the right ACC and the right FEF. The orange dots indicate the change ratio of FC between the left MFG and the left FEF. The green dots indicate the change ratio of FC between the right MFG and the right FEF. Vertical axes indicate the change ratio of FC. Horizontal axes indicate the amplitude of after**-**effect.

Fig. 5 shows the correlation between change ratio of FC and the amplitude of after-effect in the following pairs the following pairs: the right primary motor cortex and the left primary motor cortex; the right dentate nucleus and the left dentate nucleus; the right primary motor cortex and the left dentate nucleus; and the left primary motor cortex and the right dentate nucleus. The blue dots indicate the change ratio of FC between the right M1 and the left dentate nucleus. The red dots indicate the correlation between the left M1 and the right dentate nucleus. The orange dots indicate the change ratio of FC between the left M1 and the right M1 (*r=0.48, p<0.05, Pearson correlation coefficient). The green dots indicate the change ratio of FC between the left dentate nucleus and the right dentate nucleus. Vertical axes indicate the change ratio of FC. Horizontal axes indicate the amplitude of after**-**effect.
