# Supplementary material for: Correlation between changes in functional connectivity in the dorsal attention network and the after-effects induced by prism adaptation in healthy humans: A dataset of resting-state fMRI and pointing after prism adaptation
Source: Data Brief. 2018 Dec 18;22:583–9. doi: 10.1016/j.dib.2018.12.053 (PMC6322076; doi:10.1016/j.dib.2018.12.053)
Supplement: Supplementary file 1 — Supplementary material [file mmc1.pdf]

AUTHOR DECLARATION

Journal title: Data in Brief **No:** DIB-D-18-03111 R1

Manuscript title: Correlation between changes in functional connectivity in the dorsal attention network and the after-effects induced by prism adaptation in healthy humans: A dataset of resting-state fMRI and pointing trials after using prism adaptation.

We wish to confirm that there are no known conflicts of interest associated with this publication and there has been no significant financial support for this work that could have influenced its outcome.

We confirm that the manuscript has been read and approved by all named authors and that there are no other persons who satisfied the criteria for authorship but are not listed. We further confirm that the order of authors listed in the manuscript has been approved by all of us.

We confirm that we have given due consideration to the protection of intellectual property associated with this work and that there are no impediments to publication, including the timing of publication, with respect to intellectual property. In so doing we confirm that we have followed the regulations of our institutions concerning intellectual property.

We understand that the Corresponding Author is the sole contact for the Editorial process (including Editorial Manager and direct communications with the office). He is responsible for communicating with the other authors about progress, submissions of revisions and final approval of proofs. We confirm that we have provided a current, correct email address which is accessible by the Corresponding Author and which has been configured to accept email from mizuno.katsuhiro@gmail.com.

Signed by all authors as follows:

Kengo Tsujimoto	Kengo Tsujimoto 12/11/2018
Katsuhiro Mizuno (corresponding author)	Katsuhiro Mizuno 12/11/2018.
Daisuke Nishida	Daisuke Nishida 12/11/2018
Masatoshi Tahara	Masatoshi Tahara 12/11/2018
Emi Yamada	Emi Yamada 12/11/2018
Shiori Shindo	Shiori Shindo 12/11/2018
Yuuki Watanabe	Yuuki Watanabe 12/11/2018
Shoko Kasuga	Shoko Kasuga 12/12/2018
Meigen Liu	Meigen Liu 12/12/2018
